# p27^Kip1^ and Tumors: Characterization of *CDKN1B* Variants Identified in MEN4 and Breast Cancer

**DOI:** 10.3390/cells14030188

**Published:** 2025-01-26

**Authors:** Debora Bencivenga, Emanuela Stampone, Jahanzaib Azhar, Daniela Parente, Waqar Ali, Vitale Del Vecchio, Fulvio Della Ragione, Adriana Borriello

**Affiliations:** 1 Department of Precision Medicine, University of Campania “L. Vanvitelli”, Via Luigi De Crecchio, 7, 80138 Naples, Italy; emanuela.stampone@unicampania.it (E.S.); jahanzaib.azhar@unicampania.it (J.A.); daniela.parente@unicampania.it (D.P.); fulvio.dellaragione@unicampania.it (F.D.R.); 2 Centre National de la Recherche Scientifique, University of Montpellier, UMR9002, 141 rue de la Cardonille, 34396 Montpellier, France; waqar.ali@igh.cnrs.fr; 3 Department of Experimental Medicine, Section of Human Histology and Embryology, University of Campania “L. Vanvitelli”, Via L. Armanni 5, 80128 Naples, Italy; vitale.delvecchio@unicampania.it; 4 Department of Life Sciences, Health and Health Professions, Link Campus University, 00165 Rome, Italy

**Keywords:** p27^Kip1^, *CDKN1B*, cell cycle, cell motility, tumor suppressor gene, cancer

## Abstract

p27^Kip1^ is a key cell cycle gatekeeper governing the timing of Cyclin-dependent kinase (CDK) activation/inactivation and, consequently, cell proliferation. Structurally, the protein is largely unfolded, a feature that strongly increases its plasticity and interactors and enhances the number of regulated cellular processes. p27^Kip1^, like other intrinsically unstructured proteins, is post-translationally modified on several residues. These modifications affect its cellular localization and address p27^Kip1^ for specific interactions/functions. Several germline or somatic *CDKN1B* (the p27^Kip1^ encoding gene) mutations have been demonstrated to be associated with multiple endocrine neoplasia type 4 (MEN4), hairy cell leukemia, small-intestine neuroendocrine tumors, and breast and prostate cancers. Here, we analyzed the effect of four *CDKN1B* missense and nonsense mutations found in patients affected by MEN4 or cancers, namely, c.349C>T, p.P117S; c.397C>A, p.P133T; c.487C>T, p.Q163*; and c.511G>T, p.E171*. By transfecting breast cancer cell lines, we observed increased growth and cell motility for all the investigated mutants compared to wild-type p27^Kip1^ transfected cells. Furthermore, we discovered that the mutant forms exhibited altered phosphorylation on key residues and different localization or degradation mechanisms in comparison to the wild-type protein and suggested a possible region as crucial for the lysosome-dependent degradation of the protein. Finally, the loss of p27^Kip1^ ability in blocking cell proliferation was in part explained through the different binding efficiency that mutant p27^Kip1^ forms exhibited with Cyclin/Cyclin-dependent Kinase complexes (or proteins involved indirectly in that binding) with respect to the WT.

## 1. Introduction

p27^Kip1^ (hereafter p27) is an intrinsically disordered protein belonging to the CIP/Kip family of Cyclin-dependent kinase modulators [1,2,3]. The protein represents a critical regulator of the cellular responses to various stimuli and environmental factors, including signals that inhibit proliferation or promote growth, differentiation, and DNA damage [4,5,6,7,8,9,10].

Through a Kinase Inhibitory Domain (KID) located in its N-terminal region, p27 regulates the activity of nearly all CDK/Cyclin complexes, thereby playing a crucial role in controlling the progression of the cell cycle [1,2,3]. When p27 is localized in the nucleus, it binds to Cyclin E/CDK2, blocking the kinase activity and preventing transition from the G1 to the S phase. In turn, this can lead to G1 arrest and, in some cases, cause cells to enter a resting state (G0).

On the other hand, stimuli inducing proliferation can relieve p27 inhibition on CDK2/cyclin complexes by leading to the intramolecular phosphorylation of p27 on threonine 187 [11,12,13,14]. The phosphorylation targets p27 for degradation, thus playing a critical role in cell cycle progression and maintaining a low level of nuclear p27 during both the S and G2 phases [14,15]. p27 is also capable of binding CDK4(6)/cyclin D, but the details of this interaction are not precisely defined. It appears necessary for the assembly of CDK4 and Cyclin D in a ternary complex inside the cytosol, and for the transfer of the kinase complex into the nucleus [16]. p27 is also phosphorylated on Tyr74, and this phosphorylation enables the allosteric activation of the ternary complex CDK4/Cyclin D/p27 [16,17]. Intriguingly, the active CDK4/Cyclin D/phospho(Tyr74)p27 complex is insensitive to palbociclib, a chemotherapeutic drug that selectively inhibits CDK4/6 and that has been recently introduced in the therapy of hormone receptor-positive (HR+)/HER- breast cancer [17].

The intrinsic disorder of p27 enables the protein to bind several different partners and, in turn, allows for multiple biological roles in addition to the function of modulating CDK activity. Specifically, cytosolic p27 is able to control cytoskeletal dynamics, motility and invasiveness, vesicle trafficking, and cytokinesis [18,19,20,21,22,23,24]. Finally, p27 controls autophagy and transcription [25,26,27]. The content and functions of p27 are modulated through different mechanisms that include the control of *CDKN1B* (the gene encoding p27) expression and the rate of protein degradation. In addition, post-translational modifications (PTM) regulate the activity of p27 by modulating its stability, localization, and flexibility [2,3], as partially anticipated above.

In turn, the deregulation of p27 PTMs can significantly impact tumor cell behavior by altering the protein’s partners and functions. In addition to the PTMs at Thr187 and Tyr74 (and, with similar meaning, Tyr88/89), other phosphorylation events have been identified, including those on serine 10 and threonine 198 [28,29,30,31,32,33,34,35,36]. These modifications have been linked to protein stability [28,29,30,31,32,33], nuclear export [34,35], and cytoplasmic sequestration [36]. However, their precise roles remain partially unclear, and the identification of the responsible kinases is still under investigation. Given the critical functions of p27 in both the nucleus and the cytoplasm, cells tightly control its levels and localization. Any disruption in these regulatory mechanisms can lead to uncontrolled proliferation and increased risk of tumor formation and progression.

A noticeable p27 reduction has been observed in various human malignancies correlating with increased aggressiveness and poor outcomes. In particular, low p27 levels have been found in primary tumors, including breast, colon, lung, and prostate carcinomas; esophageal cancer; head and neck cancers; melanomas; and astrocytomas [37,38,39,40]. It has been initially hypothesized that increased proteolysis is the primary mechanism of p27 loss in human cancers [39,40]. Supporting this hypothesis, several investigations have shown that Skp2 and Cks1, two proteins involved in the ubiquitin-dependent degradation of p27, are overexpressed in human cancers [41]. In addition, the relocalization of p27 from the nucleus to the cytosol has also been linked to human cancers. Reduced p27 nuclear levels and enhanced cytosol accumulation are associated with poor prognoses in numerous tumors, including breast cancer, acute myelogenous leukemia, pancreatic cancer, and ovarian carcinoma [39,40].

Studies performed on the CIP/Kip and INK4 families (all inhibitors of CDK activity) focused on the alterations of the respective coding genes in human cancers [42]. Early investigations have indicated that INK4 members are frequently deleted/mutated in malignancies [42,43,44]. Only recently, with the advent of Next-Generation Sequencing, changes in *CDKN1B* were identified in tumors. *CDKN1B* alterations have been reported in breast cancers and prostate carcinomas [45,46,47] as well as in small intestine neuroendocrine tumors (SI-NETs), where mutations of *CDKN1B* represent one of the most frequent gene alterations [48,49]. In particular, Francis et al. found somatic *CDKN1B* small insertions/deletions in 10% of the analyzed si-NET, while over a cohort of 180 cases, an 8% frequency of heterozygous frameshift *CDKN1B* mutations was established [48]. To note, *CDKN1B* is now considered a driver gene for SI-NET, along with a specific subset of breast cancer defined as hormone receptor-positive (HR+) or luminal breast cancer (LBC) [50]. For the latter, investigations reported a mutation rate of approximately 3%, which becomes slightly higher (4.1%) in younger premenopausal women with LBC [50].

*CDKN1B* deleterious mutations have been found in hairy cell lymphoma (16% frequency), where it represents the second most frequent mutation, second only to *BRAF* [51]. Germline mutations of *CDKN1B* have also been identified as the cause of a type of multiple endocrine neoplasia, a rare autosomal dominant disease characterized by multiple endocrine cancers [52]. Since the initial study of Molatore and Pellegata [53], which linked a *CDKN1B* mutation to a specific form of MEN in rats, cases of human MEN1-like phenotype not bearing mutations in *MEN1* and *RET* genes have emerged, gradually confirming the association of *CDKN1B* with a distinct human MEN syndrome named MEN type 4 (MEN4) [54]. Overall, the reported data confirm that *CDKN1B* can be considered a bona fide tumor suppressor gene in humans, as also confirmed by studies in *Cdkn1b* ablated mice [55,56]. The observation of monoallelic inactivation of the *CDKN1B* gene alteration in several tumors, including si-NET, breast cancers, and parathyroid adenomas, suggests that *CDKN1B* might be considered a haploinsufficient tumor suppressor. The view is also consistent with findings from mouse models [56]. Finally, some *CDKN1B* variants classified as polymorphisms (i.e., c.326T>G, p.V109G) have also been proposed as prognostic factors in sporadic medullary thyroid carcinoma [57].

In conclusion, the germinal and somatic *CDKN1B* mutations reported with increasing frequency in the literature and variant databases suggest that p27 alterations may play a direct role in hormone-related diseases and cancers that are hormone-dependent or hormone-responsive. Only a few detailed functional studies, however, have been performed to unravel their pathogenetic mechanisms and mechanistically confirm *CDKN1B* as a susceptibility gene [50,58,59,60]. In this study, we focused our attention on four *CDKN1B* mutations that might affect p27 subcellular localization, PTMs, and/or protein removal. Precisely, we selected two missense and two nonsense variants. The results obtained suggest that the mutations can affect p27 interaction, resulting in phenotypic changes favoring cancer development.

## 2. Materials and Methods

### 2.1. Reagents and Antibodies

pcDNA3.1 plasmid carrying the human wild-type p27 coding sequence was kindly given by Prof. Pagano (Dpt. Biochemistry and Molecular Pharmacology, New York University Grossman School of Medicine, New York, NY, USA). The QuikChange II Site-Directed Mutagenesis Kit was obtained from Agilent Technologies (Santa Clara, CA, USA). λ protein phosphatase was obtained from Santa Cruz Biotechnologies (Dallas, TX, USA). Cycloheximide (CHX), Z-L-Leu-D-Leu-L-Leu-al (MG132, R stereoisomer), and *trans*-Epoxysuccinyl-L-leucylamido(4-guanidino)butane (E-64) were obtained from Merck KGaA (Darmstadt, Germany). Mouse monoclonal anti-p27^Kip1^ antibody was obtained from BD Transduction Laboratories (Franklin Lakes, NJ, USA) (RRID:AB_397637). The antibodies against Actin (RRID: AB_476693), CDK4 (RRID: AB_259094), and CDK2 (RRID: AB_1840625) were obtained from Merck KGaA. The antibodies against Cyclin D1 (RRID: AB_627344), CDK1 (RRID: AB_627224), Pin1 (RRID: AB_628132), LDHa (RRID: AB_2137192), and Lamin A/C (RRID: AB_10991536) were produced by Santa Cruz Biotechnology, Inc. (Dallas, TX, USA). Anti-Phospho(Ser10)p27 (RRID: AB_2552949) and anti-Phospho(Thr198)p27 (RRID: AB_2553787) were obtained from Thermo Fisher Scientific Inc. (Waltham, MA, USA). The HRP-conjugated secondary goat anti-rabbit and anti-mouse antibodies were obtained from Jackson ImmunoResearch Europe Ltd. (Cambridge, UK). The Lipofectamine 3000 was purchased from Thermo Fisher Scientific Inc.

### 2.2. Mutagenesis

The selected point mutations of *CDKN1B* were inserted into the cDNA of the WT-p27-pcDNA3.1 vector through mutagenesis by using the QuikChange II Site-Directed Mutagenesis Kit (Agilent Technologies, Santa Clara, CA, USA). The suggestions of the manufacturer were followed, and the nucleotide substitutions were verified through sequencing, as reported in Natalicchio et al. [61]. The oligo sequences used for the mutagenesis will be made available upon request.

### 2.3. Mammalian Cell Culture Growth Conditions and Treatments, Cell Transfection, and Lysis

The triple-negative MDA-MB-231 (HTL99004—RRID: CVCL_0062) and the ER+ MCF-7 (#HTL95021—RRID: CVCL_0031) human breast cancer cell lines were cultured in DMEM medium supplemented with 10% FBS, 100 U/mL of penicillin, and 100 mg/mL of streptomycin at 37 °C with 5% CO_2_. Cells were routinely tested for mycoplasma. All the experiments were performed on cells at early passages. Transfections were performed using Lipofectamine 3000, as reported in [62]. The efficiency of transfections was evaluated as reported in the Supplementary Materials (Figure S1). For the analysis of the p27 half-life, MCF-7 cells were seeded at 50% confluence and then, upon adhesion, transfected for 24 h with all the constructs. After that, cells were incubated with fresh medium containing 36 μM cycloheximide (CHX) for the indicated time intervals. p27 levels at each time point were determined through densitometry, and the reported values were calculated as fractions of p27 at time 0 h (w/o CHX). For the lysosome- or proteasome-dependent degradation of the truncated p27 HRVs compared to that of p27-WT, cells were incubated with fresh medium containing 36 μM cycloheximide (CHX). After three hours from CHX exposure, 1 μM MG132 (proteasome inhibitor) or 10 μM E64 (lysosomal calpain and cathepsin inhibitor) were added for the indicated time. Finally, the cells were harvested through trypsinization, washed twice with PBS, and total or compartmentalized (cytosol or nuclear) cell extracts were obtained, as reported in [63].

### 2.4. Mono- and Bi-Dimensional SDS-PAGE and Immunoblotting

The mono-dimensional SDS-PAGE was carried out as reported in [64]. For the bi-dimensional SDS-PAGE, the procedures described in [31] were followed. For Western blotting (WB), PVDF (0.2 μm pores) was used. Membranes were blocked for 1 h with 5% non-fat dry milk/TBS/0.05% Tween 20, and the incubation with primary antibodies was carried out under mild agitation for 2 h at room temperature (R.T.) for all the used antibodies. After four washes with TBS/0.05% Tween 20 (5 min/wash), incubation with HRP-conjugated secondary antibodies (added in accordance with the primary ones) was carried out for 1 h at R.T.

### 2.5. Cell Count and Flow Cytometry

For proliferation and cell cycle analyses, MCF-7 and MDA-MB-231 cells were seeded in 6-well multiwell plates (3 wells per condition) at 40% confluence. At cell adhesion, they were transfected by using 1 μg plasmid per well. After 48 h, the cells were harvested through digestion with Accutase and counted by using a hemocytometer. The count means (±SD) were reported as histograms, and the significances were calculated by the t-test. *p*-values < 0.05 were considered significantly different. The levels of overexpressed p27 were checked by lysing the cell pellets to obtain total extracts and loading 20 μg of each sample in SDS-PAGE. Finally, immunoblotting with anti-p27 and anti-Actin antibodies was performed.

The same transfections were performed for the analysis of cell cycle distribution. After collecting cells as above, cells were resuspended in ice-cold 70% ethanol, vortexed, and fixed by leaving them for 2 h at 4 °C. Then, the pellets were centrifuged at 300× *g* for 5 min, the supernatants were discarded, and 1 mL of propidium iodide staining solution was added to each cell pellet and left for 30′ at R.T. in the dark, as in [65]. Finally, the samples were quickly analyzed through FACS CANTO II (BD Biosciences, San Jose, CA, USA) flow cytometer and FlowJo V10 software (FlowJo LLC, Ashland, OR, USA). The cell cycle distribution was reported as percent of count events, from 3 independent experiments.

### 2.6. Scratch Wound Assay

For the wound healing tests, the assay was performed in a medium without FBS to avoid the contribution of cell growth on the scratch closure. Briefly, the cells were seeded in 12-well plates in a complete medium at high confluence. At adhesion, the culture medium was replaced with fresh medium without serum. Then, 1 h later, the transfections were performed for 48 h. After that, equally thin wounds were created on the cell layers, and the cells were photographed at 20× magnification using an inverted phase contrast microscope at time 0 (T_0_) and 5 h after wounding (T_5h_). Uncovered areas were measured by using Image J 1.53e (Java 1.8.0_172) software at both T_0_ and T_5h_. Equal amounts of the prepared extracts were subjected to SDS-PAGE/IB with anti-p27 and anti-Actin antibodies to verify p27-WT and HRVs levels and the loading, respectively.

### 2.7. Phosphatase Treatment

In total, 400 μg of total protein extracts (obtained by lysing transfected and non-transfected MCF-7 cells with RIPA buffer without phosphatase inhibitors) was divided into two samples, diluted in a phosphatase assay buffer (50 mM Tris-HCl, pH 7.5, 0.1 mM EDTA, 1 mM DTT, 2 mM MnCl_2_, and protease inhibitors) at a 1 μg/μL protein concentration, and incubated at 30 °C for 1 h with or without the recombinant λ phosphatase protein (500 U/assay). Finally, the samples were subjected to protein precipitation by incubation with 10% trichloroacetic acid (TCA) for 45 min on ice. The pellets were obtained through centrifugation for 30 min at 16,000× *g*, 4 °C. To eliminate the TCA, the protein pellets were washed twice with absolute ice-cold acetone, left to dry, and then resuspended in the rehydration buffer, pH range 3–10, for the isoelectrofocusing.

### 2.8. Immunoprecipitation and Pull-Down Assay

The immunoprecipitations of the phospho(Ser10)p27 isoform (alone or in combination with phospho(Thr198)p27) were performed as by Borriello et al. [29]. The immunocomplexes were eluted from the protein A/G beads by incubation with 100 mM Glycine HCl, pH 2.2. The inputs, eluates, and supernatants (S/Ns) of the experiments (as reported in the Results Section) were precipitated by TCA/Acetone as described above and resuspended in the rehydration buffer, pH 3–10, for the 2D SDS-PAGE/IB analysis. The densitometries of spots 0 and 2 in both inputs and S/Ns were carried out by scanning the obtained X Films and using the TotalLab CLIQS 4.0 gel image analysis software (TotalLab Ltd. 2019, Newcastle-Upon-Tyne, UK). The abundance of spot 2 corresponding to the monophosphorylated p27 isoforms was calculated as a percentage of total p27 (spot 0 + spot 2 densities, AU) in both inputs and S/Ns of phospho(Ser10) + phospho(Thr198)p27 immunoprecipitations.

MCF-7 cells transfected (or not) with p27-WT and p27 variants were lysed with RIPA buffer to obtain the total extracts, the protein concentrations were determined, and the same concentrations (1 μg/μL) were achieved by dilution with the lysis buffer. Then, 40 μL (≅ 40 μg) of each sample was treated at 90 °C for 5 min to purify exogenous and endogenous p27 (taking advantage of the p27 thermal stability). After that, the heat-treated extracts were clarified from precipitated proteins by centrifugation at 16,000× *g* for 10 min and the supernatants containing thermostable and partially purified p27 were collected and used as BAIT. As a PREY protein-containing extract (PP-c E), the total extract from asynchronous MCF-7 was used (400 μg each assay). Then, antibodies against p27 (3 μg/sample) were incubated overnight at 4 °C with each BAIT extract. The day after, the immunocomplexes were bound to protein A/G magnetic beads, and the beads were washed three times with lysis buffer. The beads were then incubated with 400 μg of PP-c E for 1 h at room temperature and centrifuged, and the supernatants (containing non-bound proteins) were removed. After three washes with RIPA buffer 0.5× containing protease and phosphatase inhibitors, the beads were dried and resuspended in the loading sample buffer 1× for the following SDS-PAGE/IB analyses. As controls, the pull-down without bait extracts (including only PP-c E, 1 h on the wheel at room temperature) and the non-related pull-down with control IgGs (carried out including p27-WT bait and PP-c E, not related pull-down, NR PD) were prepared and loaded on the same gel.

### 2.9. Statistics

All the experiments were performed in triplicate. When reported, the densitometries of the immunoreactive bands after both mono- and bi-dimensional SDS-PAGE/immunoblotting were obtained using TotalLab CLIQS 4.0 software. The experimental data were expressed as the mean ± SD. GraphPad Prism 6 software (La Jolla, CA, USA) or Microsoft Excel was used for statistical analyses and calculations. Comparisons among samples were performed using the *t*-test. *p*-values < 0.05 were indicative of a significant difference between samples.

## 3. Results

### 3.1. Selection of CDKN1B Variants and Their In Silico Analysis

**Figure 1:**
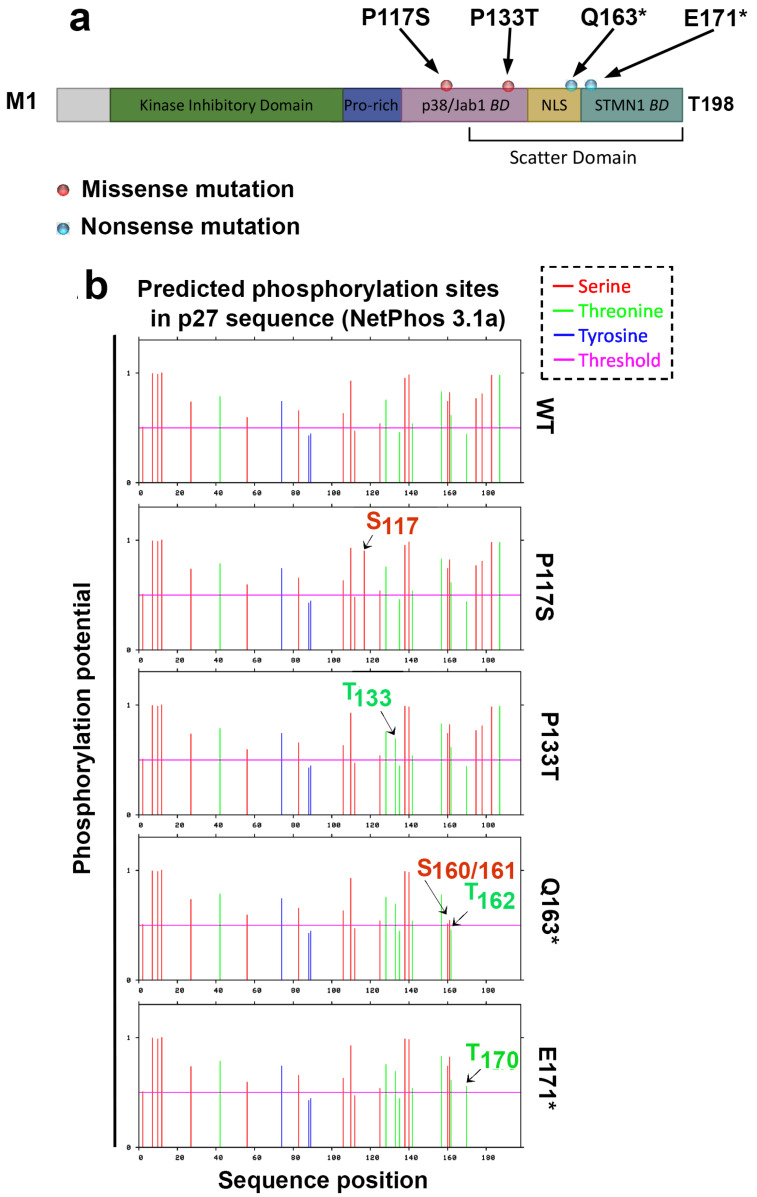
In silico prediction of serine (S), threonine (T), and tyrosine (Y) phosphorylation potential in the WT and mutated p27 sequences. (**a**) Schematic representation of p27 sequence showing the principal domains and the sites of the mutations that are the object of this study. Pro-rich, Proline-rich domain; BD, binding domain; NLS, Nuclear Localization Sequence; STMN1, Stathmin 1. (**b**) Netphos 3.1 results for the phosphorylation prediction of the p27-WT and HRVs. The arrows indicate the phosphorylation potential of the amino acids of interest.

Two missense and two nonsense *CDKN1B* variants were analyzed for mechanistic characterization. These *CDKN1B* alterations have been previously reported in the Catalogue of Somatic Mutations in Cancer (COSMIC, https://cancer.sanger.ac.uk/cosmic, last accessed on 30 September 2024) and International Cancer Genome Consortium (ICGC, https://dcc.icgc.org, last accessed on 30 September 2024) data portals, published by the National Center for Biotechnology Information (https://www.ncbi.nlm.nih.gov/, last accessed on 30 September 2024), or submitted to the public archive ClinVar (https://www.ncbi.nlm.nih.gov/clinvar, last accessed on 30 September 2024). The criteria for selecting these *CDKN1B* genetic changes were (i) their definite or possible clinical significance in human cancers and (ii) their potential effect on p27 subcellular localization and/or p27 phosphorylation. The schematic structure of human p27, with its major domains and motifs, is reported in Figure 1a, where the sites of the genetic changes analyzed in this study are also highlighted.

The first of the two *CDKN1B* missense mutations involves the substitution of proline 133 into a threonine (p27-P133T, chr12:g.12718236C>Ac.397C>A). The mutation was identified in patients affected by different diseases, including (1) head and neck squamous cell carcinoma (HNSCC) [50]; (2) primary hyperparathyroidism (PHPT), autoimmune hypothyroidism, and fibrocystic breast disease [66]; (3) meningioma, papillary thyroid carcinoma, parathyroid adenoma, and, additionally, Hürthle cell adenoma, cholesteatoma, and uterine leiomyomas, thus overall describing a MEN 4 phenotype [67]; and (4) thyroid carcinoma (somatic, COSV99963856; COSM9177399; ClinVar accession: VCV000217127) [60,68]. In some of these cases, germline alterations were identified, namely, in one patient reported in [60] and others in [66,67]. The 133 codon was also altered by a somatic nucleotide substitution (c.399A>C, p.P133=; COSV99963967) in a patient with ovarian carcinoma [69]. The second *CDKN1B* missense mutation originated from a p27 variant in which proline 117 was replaced by a serine (p27-P117S, chr12:g.12718188C>T, c.349C>T). This variant is present in population databases (rs754936421, gnomAD 0.007%) and is currently classified as a variant of uncertain significance. *CDKN1B* c.349C>T has been found in patients with MEN4 and hereditary cancer predisposition syndrome (ClinVar variation ID: 493111). In addition, Singeisen et al. (2023) reported a case of a germline c.349C>T mutation in a 54-year-old woman also diagnosed with primary hyperparathyroidism and macroprolactinoma who underwent a total thyroidectomy for multifocal papillary thyroid carcinoma. In the absence of mutations in the *MEN1* gene, a diagnosis of MEN4 was given [70]. The same variant was reported in a subject showing skin carcinoma (histology subtype: Merkel cell carcinoma), but the zygosity, somatic status, and loss of heterozygosity (LOH) details were unknown [71]. Both selected missense variants replace two highly conserved prolines within the p27 Jab1/CSN5 binding domain (positions 117 and 133). Jab1/CSN5 is a component of the COP9 signalosome complex and promotes cell proliferation by inducing p27 translocation from the nucleus to the cytoplasm [72]. Thus, the two substitutions may result in modified p27 phosphorylation and/or a change in its subcellular distribution.

The other two *CDKN1B* mutations are nonsense single nucleotide changes that lead to truncated protein variants. The first variant, p27-Q163* (chr12:g.12871770C>T, c.487C>T), has been identified in two patients with estrogen receptor-positive (ER+) breast cancer [73] and in a further patient diagnosed with invasive urothelial carcinoma (bladder cancer) (COSMIC ID: COSM1299114; ICGC ID: MU4716948) [74]. The second truncating mutant, namely, p27-E171* (chr12:g.12871794G>T, c.511G>T), was found in two patients with ER+ luminal breast cancer [73,74], in a subject with large intestine adenocarcinoma [75], and in patients diagnosed with sporadic SI-NET [76] or bladder (invasive urothelial) cancers (COSMIC ID: COSM3688044; ICGC ID: MU138318). Both gene mutations originate from a premature stop codon (at residue 163 or 171, respectively), determining the synthesis of truncated p27 proteins lacking different C-terminal sequences. The p27 C-term, which does not acquire a definite tertiary structure, is involved in numerous interactions and activities, the majority of which are independent of CDK modulation. In particular, it contains a Nuclear Localization Signal (NLS, residues 153–169). Moreover, threonine 187 phosphorylation is essential for nuclear p27 ubiquitin-dependent degradation. The C-term also includes threonine 198, the last residue of the p27 sequence, which plays a key role in maintaining protein stability and regulating the cytoskeleton [23,33] and other sites of phosphorylation by different identified kinases (such as AKT, RSK1, Citron kinase, AMPK, and Pim). It has also been directly implicated in interaction with the MT acetylating enzyme αTAT1 (aa 89–198) [20,21] and PRC1 protein, involved in MT bundling (regions 190–198) [22]. The characteristics of the four *CDKN1B* mutations and the relevant literature references are summarized in Supplemental Table S1 (ST1). Finally, the selected variants appear specifically relevant in endocrine and/or hormone-responsive tumors and, thus, hereinafter, they might be defined, on this basis, as p27 hormone-related variants (p27 HRVs).

Supplemental Table S2 (ST2) reports some biochemical features of the variants including the pI and molecular weight. In addition, the pIs for the monophosphorylated isoforms were reported to show the effect of changes in phosphorylation on the pI. These data were obtained using Expasy tools (https://web.expasy.org/compute_pi/) last version released by the Swiss Bioinformatics Resource Portal on 1 July 2020, starting from the human p27 sequence (UniProt accession number P46527).

Preliminarily, an in silico analysis was performed on these p27 HRVs using Netphos 3.1 (http://www.cbs.dtu.dk/services/NetPhos/ (accessed on 10 January 2017) to identify new phosphorylation sites or verify other potential alterations in protein PTMs. Figure 1b reports the results of a predictive analysis of p27-HRV phosphorylation in comparison to p27-WT. The prediction scores (on a scale from 0 to 1) revealed that both substitutions, P117→S and P133→T, may introduce a putative site of phosphorylation (namely, serine 117 and threonine 133, respectively). P133→T change may also affect the sumoylation of K134, which has been reported as a mechanism of response to TGF-β treatment [77]. The sumoylation might inhibit p27 interaction with CDK2, reducing the inhibitory effect of p27 on CDK2. K134 is also a site of p27 ubiquitinylation, and, thus, P133->T might interfere with p27 ubiquitin/proteasome-dependent degradation [78].

Regarding the p27 truncated variants, in addition to the obvious absence of the two major phosphorylation sites (Thr187 and 198), the in silico prediction suggested reduced phosphorylation on Ser160 and Ser161, two residues reported to be phosphorylated by GSK-3 [79]. Both mutants totally or partially lack NLS (153–169) and regions of interaction with proteins that regulate cytoskeleton dynamics, including small GTPases (Rac and RhoA), citron kinase, and stathmin [80,81,82,83,84,85]. Thus, these truncated variants might cause alterations in cell motility and cancer cell invasiveness. The p27 C-terminal domain also interacts with the MT acetylating enzyme αTAT1 and the PRC1 protein involved in microtubule bundling. Importantly, the p27-E171* truncated variant has been previously investigated [19,23,86,87,88,89] as a p27 deletion mutant useful for understanding the functions associated with the last 28 amino acids of the protein. A longer half-life, inducing reduced microtubule stability and the partial inhibition of kinase activities compared to p27 null cells, was reported [16,20,78,79]. In the present study, p27-E171* was investigated in comparison with p27-Q163* to explore aspects not covered in earlier research.

### 3.2. Phenotypes Correlated to CDKN1B Variants: Studies on Cell Growth and Motility 

**Figure 2:**
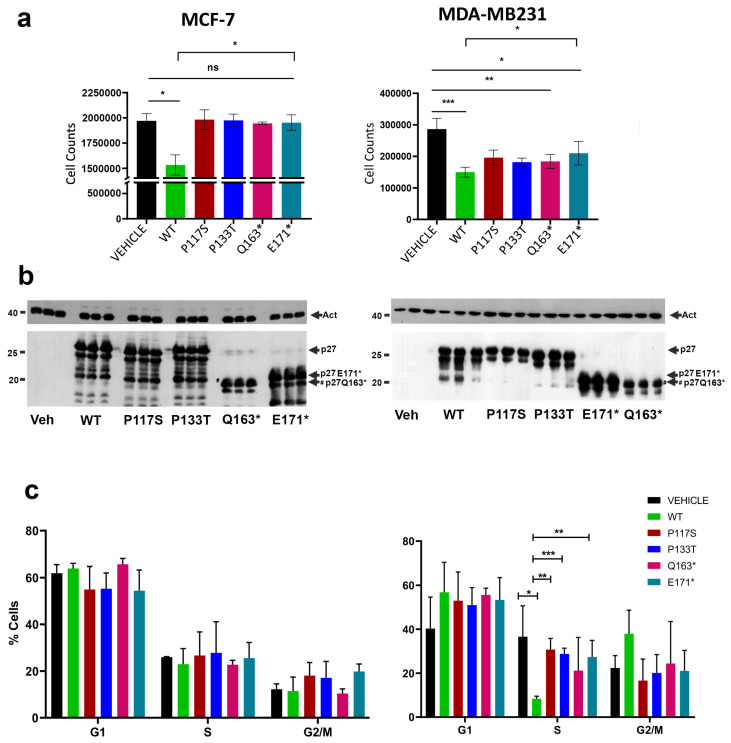
Analysis of proliferation and cell cycle distribution after the transfections of MCF-7 (left) and MDA-MB-231 (right) cell lines. (**a**) The counts of cells transfected with p27 HRV constructs were compared with those of cells transfected with p27-WT and cells exposed only to vehicles. ns, not significant; *, *p* < 0.05; **, *p* < 0.01; ***, *p* < 0.001. (**b**) The counted cells (in triplicates) were collected and lysed with RIPA buffer containing protease and phosphatase inhibitors. The extracts were analyzed by Western blotting using anti-actin antibody (as a loading control) and anti-p27 mouse monoclonal antibody to verify levels of p27 in all conditions, and the performed triplicates of transfection with each vector. Molecular weight markers are reported to the left of the blots. The arrows indicate p27 signals for (i) full-length WT and missense variants, (ii) E171*, and (iii) Q163* truncated variants. (**c**) Histograms related to cell cycle distribution (mean % ± SD) obtained through FlowJo V10 software analysis of flow cytometry experiments carried out on at least 20,000 cells per condition from three independent transfections (48 h each).

Initially, our interest focused on the effects of the exogenously expressed p27 variants on cell phenotype. Therefore, expression plasmids encoding p27 mutants were prepared by inserting the specific mutation in the human p27 coding sequence cloned in pcDNA3.1 (p27-pcDNA3.1). The plasmids were transfected into two breast cancer cell models, namely, the human ER-α+ breast adenocarcinoma MCF-7 cell line and the triple-negative (ER-α^−^, HER2^−^, PR^−^) breast adenocarcinoma MDA-MB-231 cell line. MDA-MB-231 cells were characterized by a highly aggressive phenotype. Cells were plated at 40% confluence in a complete medium. After adhesion, they were transfected for 48 h with the different constructs. Transfection efficiency ranged from 50 to 60% (in different experiments) as estimated by confocal analysis (see Supplementary Materials and Methods and Figure S1 for further details). After transfection, cells were collected and counted. The means (±SD) of three independent experiments are reported as histograms in Figure 2a. Cells were also lysed, and total protein extracts were analyzed through immunoblotting to evaluate the levels of protein expression (Figure 2b). As shown in Figure 2a, p27-WT expression determined a significant reduction of the proliferation degree in comparison to control cells (i.e., cells transfected with the empty vector). The antiproliferative activity was particularly evident in MDA-MB-231 cells (*p* ≤ 0.001). All mutations abolished (in MCF-7) or reduced (in MDA-MB-231) p27 cell growth inhibitory capacity.

The effects of the p27 HRVs on the cell cycle were also investigated by flow cytometry. As shown in Figure 2c, in MCF-7 cells, p27 variants did not appear to significantly modify cell cycle distribution (compared to p27-WT). In MDA-MB-231, p27-WT transfection caused a reduction of cells (expressed as a percentage of the total number) in the S phase compared to vehicle-treated cells, with a slight delay of both G1 and G2 phases, for which, however, no significance was obtained. The transfection of p27 HRVs significantly abrogated this S-phase reduction due to p27-WT. When compared to vehicles, the mutants did not determine significant variations in the cell cycle distribution.

**Figure 3:**
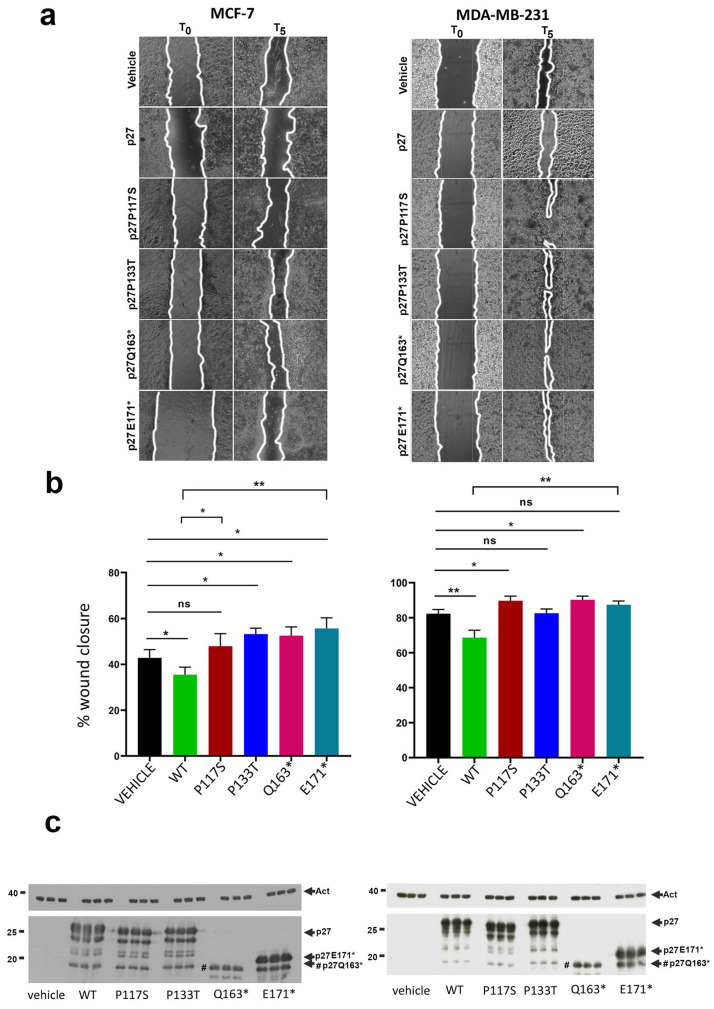
Scratch wound assays in MCF-7 and MDA-MB-231 cell lines transfected with p27-expressing vectors. Starved cells were transfected with the indicated constructs or exposed only to the vehicle. After 48 h of plasmid transfections, thin wounds were generated with p10 pipette tips; 5 h was the time empirically determined to appreciate the difference in the closure speed and, then, cell motility. (**a**) Representative images of scratch wound assays on MCF-7 and MDA-MB-231 photographed with an inverted light microscope (Leica, 10× magnification) at time 0 (T_0h_) and 5 h after inflicting the wound (T_5h_). (**b**) Histogram plots of the scratch wound assays reporting the wound closure measured using Image J 1.53e (Java 1.8.0_172) software and shown as a percentage of the initial area. (**c**) Analysis of p27-WT and p27 HRVs levels in total protein extracts obtained from cells collected at the end of scratch wound assays. Anti-actin Abs were used to control the loading. Molecular weight markers are reported to the left of the blots. The arrows indicate p27 signals for (i) full-length WT and missense variants, (ii) E171*, and (iii) Q163* truncated variants. For p values: *, *p* ≤ 0.05; **, *p* ≤ 0.01; ns, *p* > 0.05.

Subsequently, the effect of p27 variants on cell motility was evaluated by a wound healing assay performed on MCF-7 and MDA-MB-231 transfected cells. Wounds were introduced in confluent monolayers of cells transfected with an empty vector (vehicle) or p27-WT, -P133T, -P117S, -Q163*, or -E171*. To minimize the effect of proliferation over cell motility, cells were cultured in a serum-free medium, and the area of the healing was determined at time zero (T_0_) and 5 h after the scratch (T_5_). Figure 3 reports images of the two different cell populations (Figure 3a) and the histograms obtained from wound closure areas determined in three independent experiments (Figure 3b). At the end of the experiment, the cells were harvested through trypsinization, lysed, and analyzed for p27 levels through SDS-PAGE/IB (Figure 3c). As shown in Figure 3, MDA-MB-231 showed a higher capacity to heal the wounds compared to the MCF-7 cells. Both MCF-7 and MDA-MB-231 cells expressing p27-WT exhibited reduced wound closure compared to those transfected with the empty vector. In contrast, p27 mutants, including both missense and truncated variants, promoted substantial wound closure, showing that all the mutations counteracted the p27-WT negative effect on cell motility. Of interest, in the experiments performed in MCF-7 cells, the p27 HRVs’ expression determined cells’ ability to close the wounds better than that of the control cells, indirectly suggesting the activation of pathways favoring cell motility. This behavior, although less marked, was observed in MDA-MB-231 cells only when transfected with p27-P117S or p27-Q163*.

### 3.3. Subcellular Localization of the p27 Mutants

The nuclear localization of p27 correlates with cell growth inhibition. Conversely, p27 cytosolic relocalization has been frequently detected in a wide variety of tumors with a poor prognosis [90,91]. This has been explained by various mechanisms, including loss of nuclear CDK inhibition and an altered control of cytoskeletal dynamics. Furthermore, cytosolic p27 is an important factor for CDK4/Cyclin D1 complex assembly and nuclear import; thus, it might be considered a positive regulator of CDK4 kinase activity and a factor promoting cell cycle entry [92]. It was also recently demonstrated that the p27/CDK4/Cyclin D ternary complex is insensitive to the CDK4-targeting drug palbociclib, suggesting that the protein can participate in determining breast cancer palbociclib resistance [17]. Therefore, we characterized the subcellular distribution of p27 variants. These studies were carried out in MCF-7 because p27 mutations were usually correlated with luminal breast cancer and estrogen receptor-positive/HER2-negative tumors.

**Figure 4:**
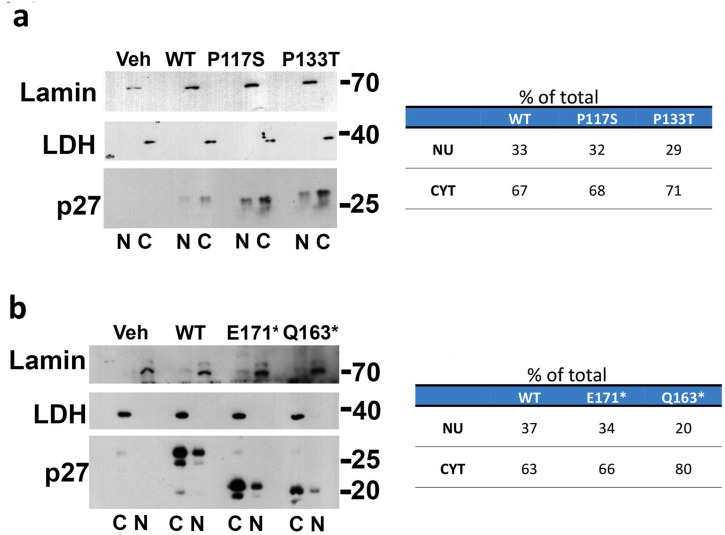
Evaluation of subcellular distribution of p27 HRVs in MCF-7 cell line. The cells were transfected for 24 h, collected by trypsinization, and washed twice with PBS. The cell pellets were then used for the compartmentalized lysis as detailed in “Section 2” and subjected to SDS-PAGE/IB analysis using antibodies against p27. LDHa and Lamin A/C were analyzed as cytoplasmic and nuclear housekeeping, respectively, and to exclude cross-contamination. The densitometry of the signals was performed. The cytoplasmic and nuclear quantities were calculated as a percentage of the total and are reported in the tables at the bottom. (**a**) Analysis performed on the missense p27 mutations compared to the WT protein. (**b**) Analysis performed on the nonsense p27 mutations in comparison to p27-WT.

MCF-7 cells were transfected with the expression vectors for 24 h and nuclear and cytosolic extracts were obtained as reported in the Materials and Methods Section. Protein extracts were then subjected to Western blotting using anti-p27 antibodies, while Lamin A/C and LDHA (LDH) antibodies were used to exclude cross-contaminations and confirm equal protein loading. As shown in the immunoblotting in Figure 4a,b, the subcellular distribution of the mutated proteins was similar to the p27-WT (~60% in the cytoplasm and ~40% in the nucleoplasm), except for p27-Q163*, which showed primarily cytoplasmic localization (80% vs. 20%), probably due to the presence of a truncated NLS.

### 3.4. Analysis of p27 Variants’ Phosphorylation

We asked whether the reduction of the antiproliferative activity and the increased capability of wound healing shown by p27 variants compared to the WT protein might be explained by or correlated with altered phosphorylation. Thus, extracts of cells transfected with p27 mutants were analyzed by 2D SDS-PAGE coupled to Western blotting with the anti-p27 antibody. We have frequently employed 2D SDS-PAGE in the characterization of p27 phosphorylation patterns since it allows for the identification of the majority of p27 phosphoisoforms and the evaluation of the ratio of phosphoisoforms/unmodified forms [29,31]. Generally, the 2D pattern of p27 shows numerous specific spots. The most basic form corresponds to the non-modified protein. This finding has been previously demonstrated by us through different approaches and confirmed by the precise correspondence between the calculated pI (by Expasy tool) and the experimentally observed value. In p27 phosphoisoform patterns reported here and in previous studies [27,29,31,58], the unmodified form is defined as spot 0 and quantitatively represents, in several asynchronous growing cell models, more than 50–60% of total p27 [29,31,93]. The monophosphorylated p27 corresponds to spot 2, as also previously demonstrated by isotopically labeled proteins [29]. A p27 isoform (spot 1) occurs between spot 0 and spot 2. It is still uncharacterized (see figure legend for additional details) and has been demonstrated to be a non-phosphorylated isoform [29]. The p27 2D pattern also displays additional acidic spots (spots from 3 to 5) that correspond to increasingly phosphorylated isoforms. A major percentage of monophosphorylated p27 corresponds to the protein phosphorylated on serine 10, while other important modifications are represented by phosphorylation on threonine 198 and, to a lesser extent, threonine 187, threonine 157, and tyrosine (74, 88, and 89), as we previously reported [31].

**Figure 5:**
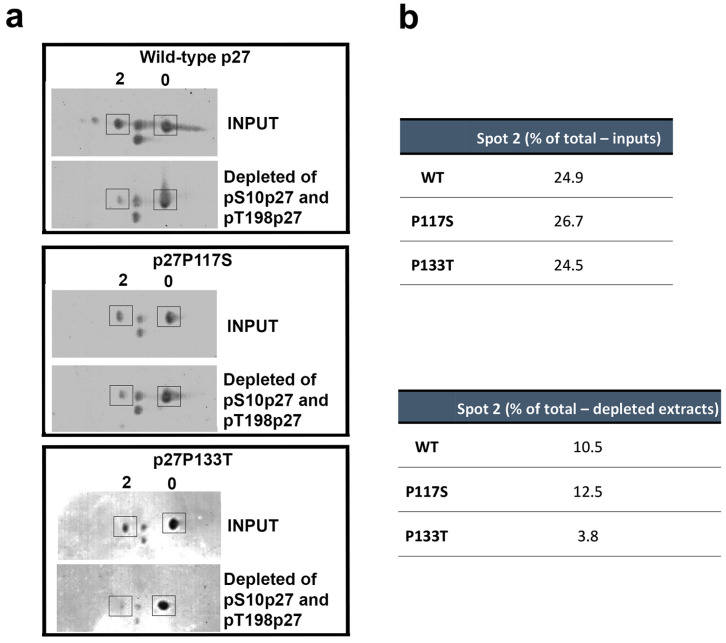
Analysis of the missense p27 HRV phosphopatterns in comparison to that of the WT protein in the MCF-7 cells. The total extracts from MCF-7 transfected with p27-WT and the two missense p27 HRVs were subjected to bi-dimensional SDS-PAGE/IB with antibodies against p27. The same extracts were used for combined immunoprecipitation of phospho(Ser10)- and phospho(Thr198)p27 isoforms by using highly specific antibodies. After the depletion of the isoforms carrying the two phosphorylated residues, the supernatants (S/Ns) of the IP were analyzed through 2D SDS-PAGE/IB with anti-p27 mouse monoclonal antibodies. (**a**) Immunoblotting of inputs (total extracts before the immunoprecipitation) and S/Ns of the performed IP with antibodies against phospho(Ser10) and phospho(Thr198) p27 (pS10p27 and pT198p27, respectively). The spot at a lower molecular weight corresponds to a putative truncated p27 (16–198 p27, expressed from another starting codon, namely, Met16). (**b**) Densitometry analysis of spot 2 (expressed as a percentage of total p27 signals) before (inputs, upper table) and after phospho(Ser10) and phospho(Thr198) isoform depletion (depleted extracts, lower table). The reported percent values were calculated with respect to the spot 0 and spot 2 intensity sum (assuming that they were the prevalent signals).

Total extracts were obtained from MCF-7 cells transfected for 24 h with p27-WT as well as with each of the p27 HRVs and analyzed by 2D SDS-PAGE/IB. Figure 5a reports the bidimensional pattern of the missense variants, namely, p27-P117S and p27-P133T, compared to p27-WT. Taking into consideration particularly spot 2 (monophosphorylated p27) and spot 0 (the unmodified protein), no remarkable differences could be evidenced in their ratio. Since the in silico analysis predicted that the mutations could insert new phosphorylatable residues in the place of P117 and P133, we re-analyzed the extracts after the removal of p27 isoforms containing phospho(Ser10) and phospho(Thr198) (the two main phosphorylated residues) by immunoprecipitation. Then, the inputs and the depleted extracts (i.e., the supernatants of the immunoprecipitations) were analyzed by 2D SDS PAGE/WB. The images were subjected to densitometry, and the abundance of spot 2 was calculated as a percentage of total p27 (spot 0 + spot 2 intensity) for each analysis. As shown in Figure 5a,b, while for p27-WT and p27-P117S, spot 2 decreased almost equally, for p27-P133T, the reduction of spot 2 was more evident, with a percentage of remnant spot 2 corresponding to 3.8% of total p27. The findings suggest that the P117S mutation did not alter the 2D phosphorylation pattern of the protein and indirectly argue against the possibility of Ser117 phosphorylation. In p27-P133T, while the intensity of spot 2 compared to spot 1 was similar to that of the WT, the remnant mono-phosphorylated isoform after phospho(Ser10+Thr198) depletion was very scarce. This finding suggests that the mutated residue was not phosphorylated and that the substitution might have enhanced the phosphorylation on both residues (Ser10 and Thr198). Further studies, mainly based on mass spectrometry, appear necessary to identify the phosphorylation sites of the p27 variants examined.

**Figure 6:**
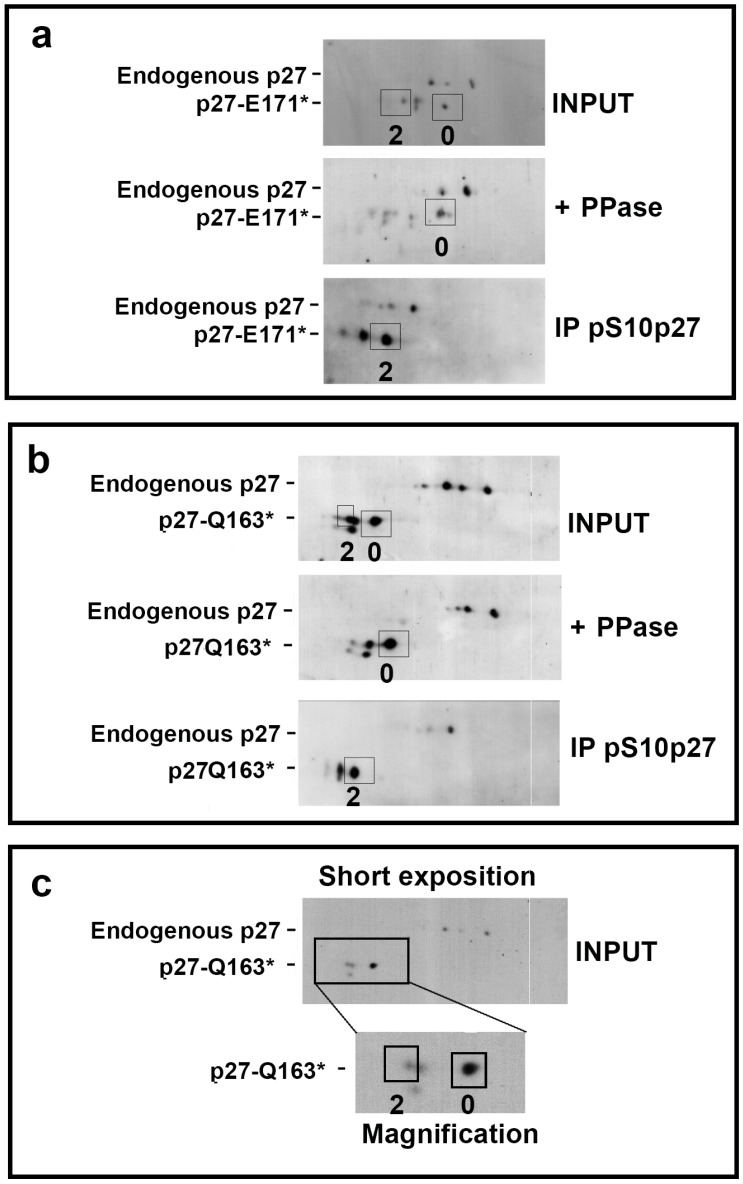
Identification of nonsense p27 HRV phosphopatterns in comparison to the endogenous protein in MCF-7. The total extracts from MCF-7 transfected with 0.1 μg of nonsense p27 HRV vectors/wells were subjected to bi-dimensional SDS-PAGE/IB with antibodies against p27. The extracts were subjected to PPase treatment and focalized. Alternatively, the same extracts were used for immunoprecipitating phospho(Ser10)p27 and the IPs were analyzed through 2D SDS-PAGE. (**a**) Analysis of p27-E171*. (**b**) Analysis of p27-Q163*. (**c**) Lower exposure of the X film related to p27-Q163*. The squares show the magnified region. The signals of endogenous p27 and overexpressed p27 are evidenced. Input, extract used as starting material for IP and as non-treated (−PPase) sample; +PPase, sample treated with λ Phosphatase.

Then, the 2D analyses of cells transfected with the truncated variants were carried out. Since truncations caused changes in the pI as well as in the molecular weight of the variants (as reported in Table S2), MCF-7 cells were transfected with a lower amount of expression vectors (1/10) than that used in the experiment reported in Figure 5 (see Section 2 for the details). This strategy allowed us to detect endogenous p27 in 2D/WB and use it as an internal control. Accordingly, we evidenced in the same blot the phosphorylation pattern of endogenous p27 and that of the transfected truncated p27 variant. The transfected extracts were also treated with recombinant λ phosphatase (PPase) for the identification of the phosphoisophorms or, alternatively, subjected to the immunoprecipitation with anti-phospho(Ser10)p27 antibodies to confirm the positioning of mono-phosphoisoforms. The anti-phospho(Thr198)p27 IP was not performed due to the absence of Thr198 in both variants. In detail, the extracts were divided into three aliquots. One aliquot was employed as a control (−PPase), one was dephosphorylated by PPase (+PPase), and the third was employed for IP with anti-phospho(Ser10)p27 antibodies (IP pS10-p27). Then, the samples (including the IP materials) were analyzed by 2D/WB with anti-p27 antibodies. As reported in Figure 6a,b, both variants showed isoforms focalizing at lower pH, according to their calculated pI (Table S2). Regarding p27-E171* (Figure 6a), the 2D pattern showed spot 2 to be of lower intensity in comparison to spot 0, different from p27-WT. We confirmed that spot 2 of the truncated variant was a phosphoisoform since we observed a spot with the same migration in phospho(Ser10)p27 IP (Figure 6a, bottom blot). Interestingly, the IP blot (Figure 6a, bottom) suggested an overall stronger phosphorylation on Ser10, with two spots having comparable intensities in the truncated p27 mutant compared to the endogenous protein. This result may have been due to the lack of many different sites of phosphorylation due to the truncation.

The same analysis was performed on the extracts from cells transfected with p27-Q163* (Figure 6b). Again, the phosphatase assay and the immunoprecipitation of the phospho(Ser10) isoform favored the identification of spot 2 (Figure 6b). Figure 6c shows a brief exposure for Q163* (−PPase and input of the IP) and the magnification of the region of interest, which clearly highlights spot 2, showing that it focalized very close to the intermediate spot, presumably spot 1, corresponding to the p27 isoform carrying an uncharacterized modification.

**Figure 7:**
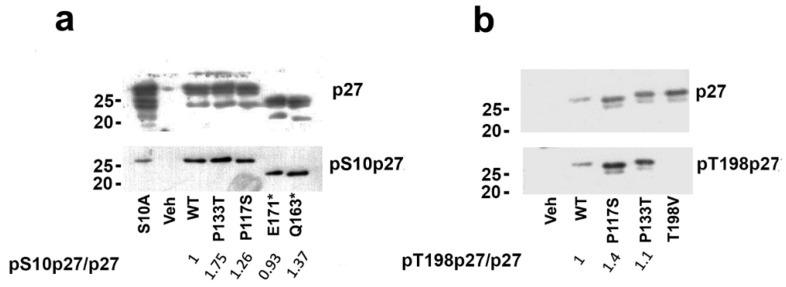
Analysis of phospho(Ser10) and phospho(Thr198) levels. MCF-7 was transfected for 24 h with the indicated constructs (1 μg/well of 6-well multiwell). The total extracts (20 μg/lane) were subjected to SDS-PAGE/IB with rabbit polyclonal antibodies against (**a**) phospho(Ser10)p27 or (**b**) phospho(Thr198)p27. After the IBs, the membranes were stripped and blotted with anti-p27 mouse monoclonal antibody. The densitometry of the immunoreactive bands was performed and the ratios of phospho(Ser10 or Thr198)p27/total p27 (pp27/p27) were calculated and are reported on the bottom of each panel.

To confirm the data obtained by the 2D/WB analysis, the content of phospho(Ser10) and phospho(Thr198) in the missense variants was investigated by 1D/WB by employing specific antibodies. The content of phospho(Thr198) in the truncated variants was not analyzed for the absence of the relative residue. The analysis was performed in MCF-7 transfected cells. In addition, specific mutants were employed to confirm the specificity of anti-phospho-p27 antisera. As shown in Figure 7a, the obtained results confirmed a relative phosphoSer10 increase in all the missense variants, particularly in p27-P133T. This could suggest that the two changed prolines could affect serine 10 phosphorylation due, for instance, to changes in the substrate affinity by the putative kinases. Alternatively, the stability of these isoforms could be increased. The higher levels of serine 10 phosphorylation agreed with the increased ability of transfected cells to migrate faster than the WT-overexpressing cells (Figure 3) and were in accordance with data provided by Li et al. [94]. The degree of the phosphoSer10 observed in Q163* was 30% higher than in the WT, probably due to the cytoplasmic sequestration of this variant (Figure 7a).

We also analyzed separately the levels of Thr198 phosphorylation. The analyses included only the missense p27 HRVs in comparison to the WT. The truncated p27 HRVs were excluded due to the loss of the C-term fragment. The dephosphomimetic mutant T198V-p27 was included as a control of the specificity of the anti-Thr198-p27 antibody. As shown in Figure 7b, we were able to exclude a consistent variation in Thr198 phosphorylation in p27-P133T. Conversely, an increase was detected for the P117S mutant in comparison to the WT (Figure 7b), suggesting that the P117→S change might modulate the efficiency of putative kinases involved in this modification or stabilize the phosphoform.

### 3.5. Analysis of Nonsense p27 HRV Degradation Mechanisms

**Figure 8:**
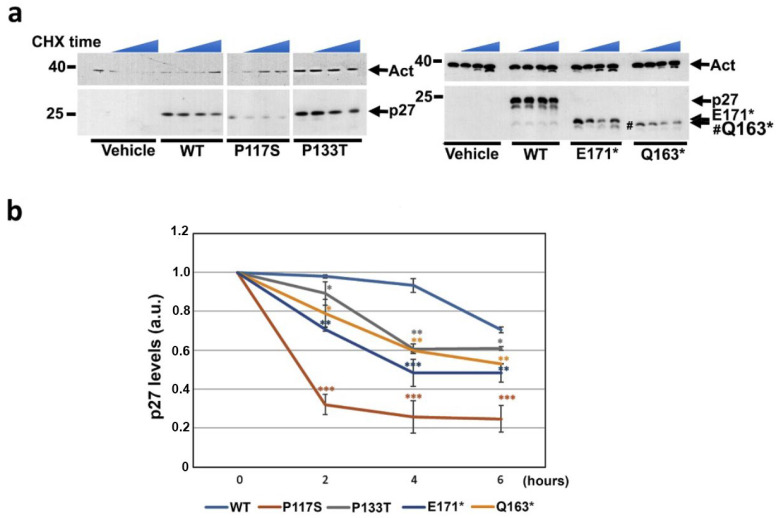
Analysis of the half-life of p27-HRVs compared to p27-WT. (**a**) The MCF-7 cells were transfected with WT or missense (left) and nonsense (right) p27 HRVs for 24 h; then, they were exposed to 36 μM CHX for 2, 4, and 6 h. At the end of each selected time, cells were collected and total extracts were prepared and analyzed through SDS-PAGE/IB with anti-p27 antibodies. Actin was analyzed as a loading control. (**b**) The immunoreactive signals obtained from three independent experiments were subjected to densitometry. The p27 level at each time point was reported as a fraction of the p27 level at time t_0_. For p values: *, *p* ≤ 0.05; **, *p* ≤ 0.01; ***, *p* ≤ 0.001.

Subsequently, we focused our attention on the degradation mechanisms of the p27 variants. This investigation might be particularly informative since one of the truncated variants (p27-E171*) lacked both the main site of nuclear phosphodegron (Thr187) and the last residue (Thr198), while the other (Q163*) also lacked, in addition to the abovementioned threonines, an integer nuclear localization signal. Several distinct mechanisms of p27 removal have been described that occur in the nucleus or cytosol and in distinct cell cycle phases [1,2,3]. The well-known nuclear degradation involves an initial phosphorylation on Thr187 followed by ubiquitin-dependent proteasome removal [14]. The process is mostly observed in S/G2 phases. A different mechanism, not yet completely unraveled, occurs in the cytosol (i.e., ubiquitination/degradation) and does not appear to require specific degradation-targeting p27 phosphorylation. Finally, lysosomal p27 degradation was reported [95], but details on this mechanism are not available. The half-life of both missense and nonsense p27 HRVs was first evaluated in comparison to that of the WT protein: MCF-7 cells were transfected for 24 h with the indicated constructs, and then CHX was added. Cells were collected at the indicated times of CHX treatment, and the total extracts were analyzed through SDS-PAGE/IB (Figure 8a). The image on the left related to the missense variants was obtained from an IB that also analyzed other variants that are not the object of this study. Therefore, the original blot is reported for transparency in Figure S2 of the Supplementary Materials. The densitometry of the p27 immunoreactive signals was performed on three independent experiments and the mean values (±S.D.), normalized on the relative actin IB, were reported as curves with respect to the p27 signal intensities at time t_0_ (fixing 1 a.u. for the starting p27 levels) (Figure 8b). As shown, the p27 HRVs (p27-P117S, in particular) exhibited significantly faster kinetics of degradation in comparison to the WT counterpart. These results might explain the loss of cell proliferation control of the mutants.

**Figure 9:**
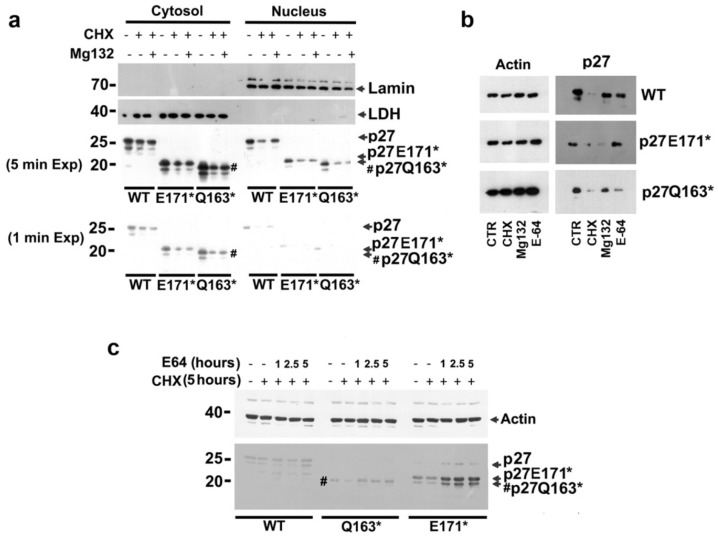
Analysis of nonsense p27 HRV degradation. (**a**) The MCF-7 cells were transfected for 24 h, as indicated; then, they were exposed to CHX for 6 h. In the last two hours of exposure, 1 μM MG132 was added. Then, nuclear and cytoplasmic fractions (30 μg/sample) were analyzed by Western blotting for p27 (1′ and 5′ exposure are reported). LDH and Lamin A/C were also analyzed as controls for efficacious separation and loading. (**b**) Cells were treated as in (**a**) except that in the last two hours of CHX incubation, MG132 (1 μM) or, alternatively, E-64 (10 μM) was added. Then, total extracts (20 μg/sample) were analyzed by SDS-PAGE/WB for p27 and actin (as loading control). CHX, cycloheximide; MG132, Z-L-Leu-D-Leu-L-Leu-al (R stereoisomer); E-64, *trans*-Epoxysuccinyl-L-leucylamido(4-guanidino)butane. (**c**) Time course experiments of MCF7 cells pretreated with CHX (for 3 h) and then exposed to cotreatment with E64 for the reported times (1, 2.5, 5 h). Actin was analyzed as loading control.

In view of the relevance of the C-term moiety of p27 for protein degradation, we analyzed the effect of nonsense mutations on the proteasome-dependent p27 degradation in both the nucleus and the cytoplasm. The cells transfected with the truncated p27 HRVs and p27-WT were treated for 6 h with cycloheximide (CHX, 36 μM), an inhibitor of protein synthesis. In some samples, during the final 2 h of CHX treatment, a potent membrane-permeable proteasome inhibitor (MG132, 1 μM) was added. The cells (CTRL and those treated with CHX and CHX+MG132) were subsequently subjected to a compartmentalized lysis. The nuclear and cytoplasmic fractions (20 μg/sample) were then analyzed for the content of p27 (or its variants). Lamin A/C and LDHa were also investigated to confirm nuclear/cytoplasmic fractionation efficacy. The data reported in Figure 9a show a different distribution of the truncated proteins, with a major quota in the cytoplasm (as also reported in Figure 4b). When MG132 was added to the CHX-exposed cells, a strong nuclear increase of p27-WT was observed, confirming that the phospho(Thr187)-dependent nuclear degradation was the prevalent mechanism controlling the WT protein homeostasis. On the other hand, no relevant accumulation of the mutant proteins was observed, except for a faint cytoplasmic increase observed for Q163*. The results indicated that the two variants, and particularly p27-E171*, were barely sensitive to proteasome degradation, and the involvement of lysosome degradation was investigated. In this case, the transfected cells treated with CHX (36 μM) were exposed to E-64 (10 μM), chosen as a lysosomal calpain and cathepsin inhibitor. The treatment with MG132 (1 μM) was also included for comparison (Figure 9b). Interestingly, while p27-WT was increased in both treatments, the two truncated p27 HRVs showed divergent pathways for their removal. In fact, Q163* levels were partially restored under MG132 exposure only. Conversely, E171* accumulated only upon E-64 treatment (Figure 9b), suggesting lysosomal proteases as major effectors in the mechanism of E171* degradation. Accordingly, the region between 163 and 170 amino acids might be required for targeting the proteins to the lysosomes. The effect of the inhibition of lysosomal-dependent degradation was evaluated in the experiment reported in Figure 9c. Cells were transfected and then treated with CHX. After 3 h, E-64 was added and cells were collected at 1, 2.5, and 5 h. The total extracts were then analyzed. As shown, while p27-WT was scarcely affected, the levels of p27-E171* and p27-Q163* (to a very limited extent) were restored by cotreatment with E64. Overall, these results suggest that the truncated variants, and in particular p27-E171*, were degraded at the lysosomal level.

### 3.6. Effects of Mutations on p27 Interactions

**Figure 10:**
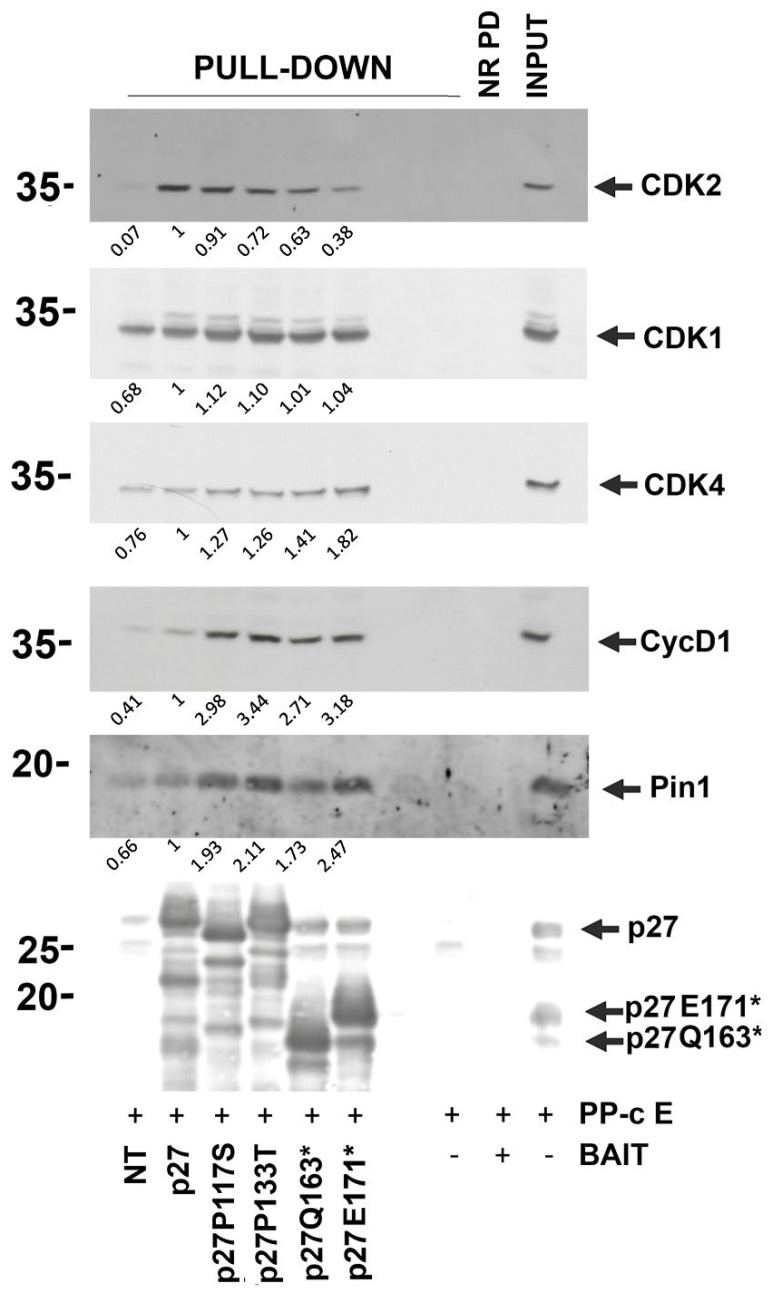
Analysis of p27 HRV interactions. In total, 40 μg of heat-treated MCF-7 transfected cells were used to prepare the BAITs (p27 WT and p27 HRVs) in the pull-down experiment. They were blocked on protein A/G magnetic beads through anti-p27 antibodies. Three washes with RIPA buffer were performed to exclude non-specific bound thermostable proteins remaining in the BAIT extracts. Asynchronous MCF-7 cells in the exponential phase of growth were lysed to obtain the total extract, and 400 μg was used for each pull-down as a source of p27 prey (PP-c E, prey protein-containing Extract). The interaction was performed by 1 h of incubation at room temperature on a wheel; after that, three washes were performed with RIPA buffer, and the beads (with immobilized immunocomplex and stable interactors of the BAITs) were resuspended in loading sample buffer for the SDS-PAGE/IB with the indicated antibodies. The pull-down was also performed by using as a BAIT 40 μg from non-transfected cells to verify the contribution of the endogenous protein (first lane). The last three lanes represent, respectively, (i) the pull-down without BAIT, (ii) the non-related pull-down (without Abs anti-p27 and with p27 WT BAIT and PP-c E) (NR PD, non-related pull-down), and (iii) PP-c E (INPUT, 30 μg).

An important point to be addressed in evaluating the effects of p27 mutations was the possible alteration of p27 interactions. To avoid eventual artifacts due to different subcellular localization and not to modified interactions, we preferred to use in these experiments the pull-down assay technique and not immunoprecipitation. So, transfected and non-transfected MCF-7 cells were lysed with RIPA buffer to obtain the total extracts. Then, 40 μL (≅40 μg) of each sample was treated at 90 °C for 5 min to roughly purify p27 (taking advantage of the thermal stability of the protein). After that, the heat-treated extracts were centrifuged at 16,000× *g* for 10 min, and the supernatants enriched by p27-WT (or its variants) were collected and used as BAIT. In parallel, exponentially growing MCF-7 (without any manipulations) was lysed using RIPA buffer and used in the pull-down as the source of p27-interacting proteins (in Figure 10, it is indicated as PP-c E, prey protein-containing extract). The first step was the overnight incubation of bait extracts with p27 antibodies. Then, the immunocomplexes were bound to protein A/G magnetic beads. Subsequently, 400 μg of PP-c E was added to the packed beads at room temperature. After 1 h of incubation, the beads were washed, and equal volumes of loading sample buffer were added to the elutions (for further details, see Section 2). Finally, the eluted samples were analyzed by Western blotting with antibodies against p27 and putative p27 interactors. The focus of this study was primarily on CDK complexes. As controls of the experiments, the pull-down without bait extract (including only PP-c E) and the non-related pull-down with control IgGs were prepared and loaded. The former was used to exclude the signals in the pull-down assays due to the endogenous p27 partners in the PP-c E and the latter was used to exclude non-specific interactions between the protein A/G beads and bait and/or prey. The levels of CDK2, CDK4, Cyclin D1, CDK1, and Pin1 bound to p27-WT or its variants were investigated. As shown in Figure 10, the major efficiency in binding CDK2 was recorded for the WT bait; conversely, it decreased in the investigated variants, achieving the lowest level for the E171* bait. These results highlighted that the HRVs p27 mutants were rarely CDK2 interactors. This was not observed for CDK1, for which a slight increase in the binding of the analyzed variants compared to the WT protein could be detected. On the other hand, p27 mutants were able to bind to CDK4 and Cyclin D1 at a higher level compared to the WT. Considering the role of p27 in forming CDK4/Cyc D complexes and favoring nuclear import (a well-known role in contrast to its oncosuppressive functions) and activation, these observations can explain the hyperproliferative phenotypes of cells expressing the variants.

Finally, we focused our attention on p27 interaction with Pin1. Pin1 is a peptidyl-prolyl *cis-trans* isomerase able to bind p27 when phosphorylated on Ser10 or, mainly, Thr187 [96]. The enzyme might change the pSer/pThr peptidyl-Prolyl cis-trans isomers, relieving p27-dependent CDK2 inhibition. Thus, an increased interaction between p27 and Pin1 might enhance CDK2 activity, favoring cell proliferation. As reported in Figure 10, all the investigated p27 mutants were able to bind Pin1 to a major extent compared to p27-WT. Of particular interest, the P133T variant showed the most binding with the isomerase, probably as a consequence of the phospho(Ser10) content.

## 4. Discussion

Key structural features of p27 are its remarkable flexibility and the absence of a stable 3D structure. These characteristics allow the protein to have numerous interactors and, thus, modulate many important processes. Recently, it has also been proposed that p27 can act as a scaffold where various complexes assemble [2]. The lack of a definite structure is, on the other hand, partially balanced by the occurrence of several p27 PTMs that reduce the flexibility and challenge the protein for specific interactors and, thus, the control of particular processes. Similarly, changes in subcellular distribution can modify p27 interactors, thus shifting p27 activities. In this study, we investigated the effects of four *CDKN1B* mutations identified in human diseases that have the common characteristic of being dependent on or sensitive to hormones. We analyzed whether *CDKN1B* genetic changes might affect PTMs, cellular distribution, and interactors of p27.

Two of the selected mutations shared the possibility of changing the phosphorylation pattern of the corresponding mutated proteins by introducing two novel phosphorylatable residues (Ser117 and Thr133) in place of the two corresponding prolines present in the WT protein. The other two investigated genetic changes caused a premature truncation of the p27 sequence, forming a protein of 162 or 170 residues instead of the 198 aa sequence of human p27. The truncation resulted in the loss of two phosphorylatable residues (Thr187 and Thr198) and the absence of additional functional domains, as detailed above in the text. Considering that *CDKN1B* is mutated predominantly in luminal breast cancers [46], we analyzed the phenotypic effects (i.e., alterations of cell proliferation, cell cycle, and motility) associated with p27 HRV overexpression in two breast cancer cell models, the (ER+PR+/-HER2^−^) MCF-7, and the triple-negative MDA-MB-231 cell lines. We found that the overexpression of p27 HRVs in the hormone-responsive model (MCF-7, luminal subtype with low Ki67) resulted in the loss of p27 antiproliferative activity. In MDA-MB-231 cells, p27 HRVs showed a reduced ability to block cell proliferation (in comparison to the WT transfected counterpart), although they kept the ability to reduce proliferation when compared to vehicle-treated cells. When we focused on cell motility, we surprisingly found that MCF-7 cells overexpressing all p27 HRVs displayed a significant increase in comparison to cells expressing WT-p27. Conversely, in MDA-MB-231, a limited positive effect was determined, with a faint increase in wound healing observed only for P117S and Q163*. As detailed in paragraph 3.1, p27-E171* has been the object of previous studies, mainly as a tool to study the roles of the C-term of the protein, particularly the Thr198 final residue and its phosphorylation [19,23,86,87,88,89]. Our results are in accordance with the studies that reported the defects of the mutant in inhibiting the motility/invasivity capacity of glioblastoma cells and tumor-induced neoangiogenesis *in vivo.*

The analysis of p27 variant PTMs suggests that both p27-P133T and p27-P117S present increased phosphorylation on Ser10 compared to their normal counterparts. Particularly, p27-P133T showed among the p27 HRVs the highest level of Ser10 phosphorylation. p27-P117S also, besides a slight increase in phospho(Ser10), showed augmented T198 modification (Figure 7). When the p27 forms were analyzed upon subtraction (by immunoprecipitation) of the phosphoisoforms modified in Ser10 and Thr198 (Figure 5), a signal for the 1P form was still evident, particularly for the P117S variant. However, in both cases, the ratio of 1P/UM form was not clearly different from that observed in p27-WT, preventing us from concluding that the sites of changes had become sites of phosphorylation. However, studies by mass spectrometry might be necessary to demonstrate the PTMs present in these variants. Both the truncated variants lacked the phosphorylatable sites threonine 187 and 198. Accordingly, they retained serine 10 as the major phosphorylation site. In particular, p27-Q163* had higher phosphorylation in position 10 compared to p27-E171*. Precisely, Ser10 phosphorylation is increased in three out of four p27 HRVs analyzed. It is important to underline the role of Ser10 phosphorylation in the nucleus–cytoplasm shuttling and cytoplasmic p27 functions, particularly the control of cell motility. Altogether, these results confirm that the contribution of phosphoThr198 in influencing cell scattering is lower than that dictated by the phosphorylation of Ser10. The two truncated variants also show a distinct subcellular localization, with p27-Q163*, which lacked the integer NLS domain, showing definite cytosolic accumulation. The nucleus/cytosol distribution of the other variant was, on the contrary, similar to that of the wild-type protein.

The investigation of p27 variant degradation suggests that in specific instances, mutations can affect the mechanism of protein removal. As a matter of fact, a shift from proteasomal degradation to lysosomal removal was observed for the p27-E171* variant. Since p27-Q163* seems to still rely on proteasome as a major mechanism for removal, it is possible to suggest that the sequence between 163 and 171 includes a consensus for lysosomal p27 degradation. We can speculate that a lysine in position 165, lost by the stop mutation at 163 and described as a site of polyubiquitination, might be involved in this process, being conserved in p27-E171*.

A major achievement of this study is regarding the interactions of the p27 mutants compared to the wild-type protein. Some p27 interactors were selected based on their ability to regulate cell growth and motility. As shown in Figure 10, all four p27 variants bound less CDK2, suggesting that they were unable (or less capable) to inhibit this CDK. In this way, CDK2 was more active, accelerating the overcoming of the G1->S transition. Intriguingly, the effect of low CDK2 binding was most evident in the truncated proteins, suggesting that the absence of the C-terminus negatively affected the interaction with CDK2. Conversely, the interaction of p27 variants with Cyclin D1 and CDK4 appeared to increase compared to p27-WT. The finding is extremely interesting since it is well known that p27 is required for Cyclin D1 binding and activation of CDK4 [16,17]. Thus, the result suggests that the variants contemporaneously favor CDK4 activation and have minor inhibitory activity on CDK2. These results might explain (at least in part) the loss of antiproliferative activity of the analyzed p27 HRVs. The effect of increased Pin1 interaction is also of interest. The enzyme allows the *cis*/*trans* isomerization of peptide bonds between phosphorylated Ser/Thr and prolyl residues. It has been reported that the peptidyl–prolyl *cis/trans* isomerase interacts with p27, reducing its binding with CDK2 [96]. This finding suggests that the enhanced association of p27 mutants with Pin1 might also be a factor explaining the reduction of p27 interaction with CDK2.

In conclusion, we observed that for all the investigated p27 variants associated with hormone-related human cancers, there was a loss (complete or partial) of antiproliferative activity and an increase in motility. The effects on protein metabolism and localization seem to depend primarily on the specific mutations and the absence (or presence) of NLS. Importantly, our results on the E171* variant may reveal the importance of a specific region (p27 residues 163–170) to address the protein involved in lysosomal (other than proteasomal) degradation, at least in conditions of altered protein structure. A major achievement of this study is the finding that the mutations strongly affect p27 interactome. In particular, their binding with CDKs or proteins that modulate p27/CDK2 binding (i.e., Pin1) was clearly altered. Of note, the decrease in p27 binding to CDK2 was, at least in the mutations investigated, associated with an increase in binding to CDK4.

Through our analysis of p27 interactions, we identified several common characteristics that serve as a unifying thread across all p27 HRVs. These features persist even though the amino acid alterations occur outside the KID and independently of the affected domains. This underscores the necessity for further comprehensive studies of p27 mutations that encompass a broad spectrum of analyses.

## Data Availability

The raw data of the experiments reported in this paper have been uploaded to the MDPI repository and/or will be made available by the corresponding author: adriana.borriello@unicampania.it.

## Supplementary Materials

The following supporting information can be downloaded at: https://www.mdpi.com/article/10.3390/cells14030188/s1, Figure S1: Immunofluorescence images of cells transfected with p27 WT, missense, and nonsense variants; Figure S2: Original blots for figure 8a (left blots); Table S1: *CDKN1B* variants analyzed in the study; Table S2: Calculated PI and MW of the p27 variants analyzed in the study.
