# Supplementary material for: p27Kip1 and Tumors: Characterization of CDKN1B Variants Identified in MEN4 and Breast Cancer
Source: Cells. 2025 Jan 26;14(3):188. doi: 10.3390/cells14030188 (PMC11817124; doi:10.3390/cells14030188)
Supplement: Supplementary file 1 [file cells-14-00188-s001.zip › cells-3341448-supplementary.docx]

*Supplementary Material*

**p27^Kip1^ and tumors: characterization of *CDKN1B* variants identified in MEN4 and breast cancer**

**Debora Bencivenga^1^**^, Emanuela Stampone^1^^, Jahanzaib Azhar^1^, Daniela Parente^1^, Waqar Ali^2^, Vitale Del Vecchio^3,4^, Fulvio Della Ragione^1^, and Adriana Borriello^1^**

^1^ Department of Precision Medicine, University of Campania "L. Vanvitelli", Via Luigi De Crecchio, 7, 80138, Naples, Italy.

^2^ CNRS - University of Montpellier UMR9002, 141 rue de la Cardonille, 34396 Montpellier, France.

^3^ Department of Experimental Medicine, Section of Human Histology and Embryology, University of Campania "L. Vanvitelli", Via L. Armanni 5, 80128 Naples, NA, Italy.

^4^ Department of Life Sciences, Health and Health Professions, Link Campus University, Rome, 00165, Italy.

Correspondence: * (A.B.) [adriana.borriello@unicampania.it](mailto:Adriana.borriello@unicampania.it); **(D.B.) [debora.bencivenga@unicampania.it](mailto:debora.bencivenga@unicampania.it)

^ These authors share the first-authorship

**Supplementary Table S1: *CDKN1B* variants analyzed in the study**


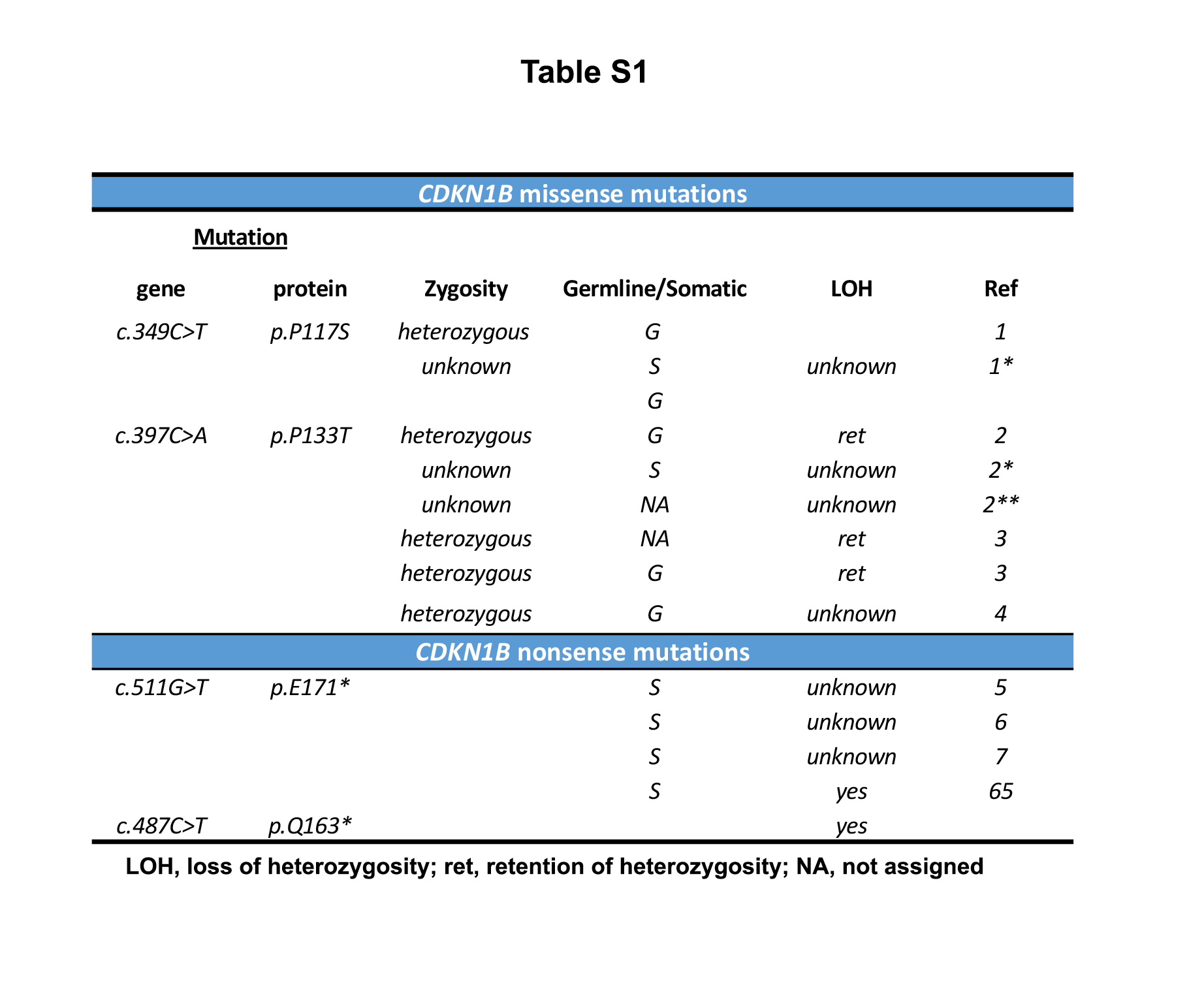


**Supplementary Table S2: Calculated PI and MW of the p27 variants analyzed in the study**

**
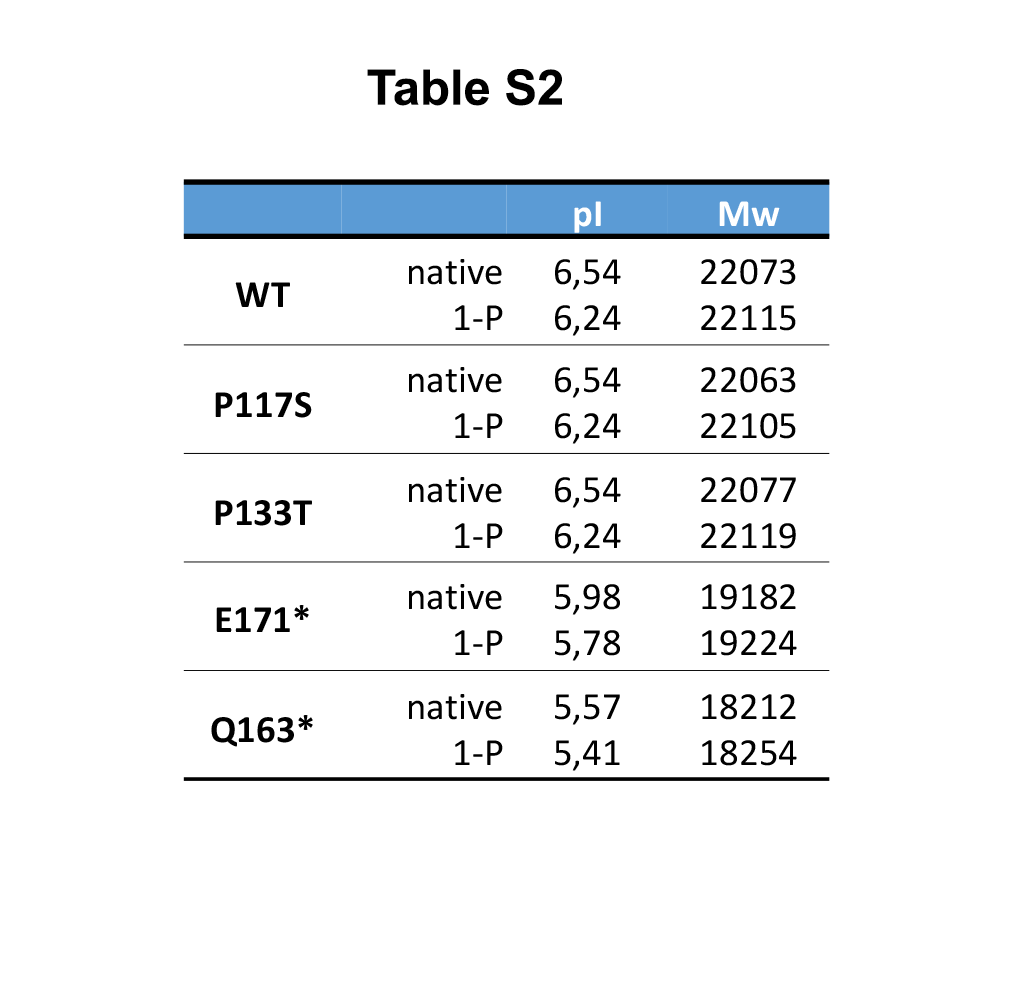
**

**S1. Evaluation of transfection efficiency**

The efficiency of transfection was verified by immunofluorescence. Briefly, MDA-MB-231 and MCF-7 cells were grown on coverglasses. At the adhesion, they were transfected with the indicated plasmids for 24 hours. Finally, they were processed for immunostaining (with anti-p27 mouse monoclonal/AlexaFluor 488 goat anti-mouse secondary antibodies), and the staining of DNA (Hoechst) as reported in Bencivenga *et al*. [Ref. S1, or Ref. 55 in the main manuscript]. The images were obtained using a Carl Zeiss (Oberkochen, Germany) LSM 700 confocal laser scanning microscope and three images from each slide were selected randomly for imaging. The randomly selected fields from three independent experiments were used for the calculation of transfection efficiency, by counting by eyes transfected cells (showing the highest green fluorescence) and total cells (Hoechst-positive cells). Then, the efficiency was expressed as the percent of transfected cells on the total cell number. Transfection efficiency ranged from 50 to 60% (in different experiments) as estimated by confocal analysis. **Figure S1** is illustrative of cells transfected with p27-WT and all the HRVs in both MCF-7 and MDA-MB-231.

The Materials used for the supplemental information are reported in paragraph 2.1 of the main text. Furthermore, the AlexaFluor 488-conjugated goat-anti-mouse secondary antibody (RRID: AB_2576208) and the Hoechst 33342 staining dye solution were used (Abcam, Cambridge, UK) and the Alexa Fluor™ 555 Phalloidin was also utilized to mark microfilaments (ThermoFisher Scientific Inc., Waltham, MA, USA).

**Figure S1.**


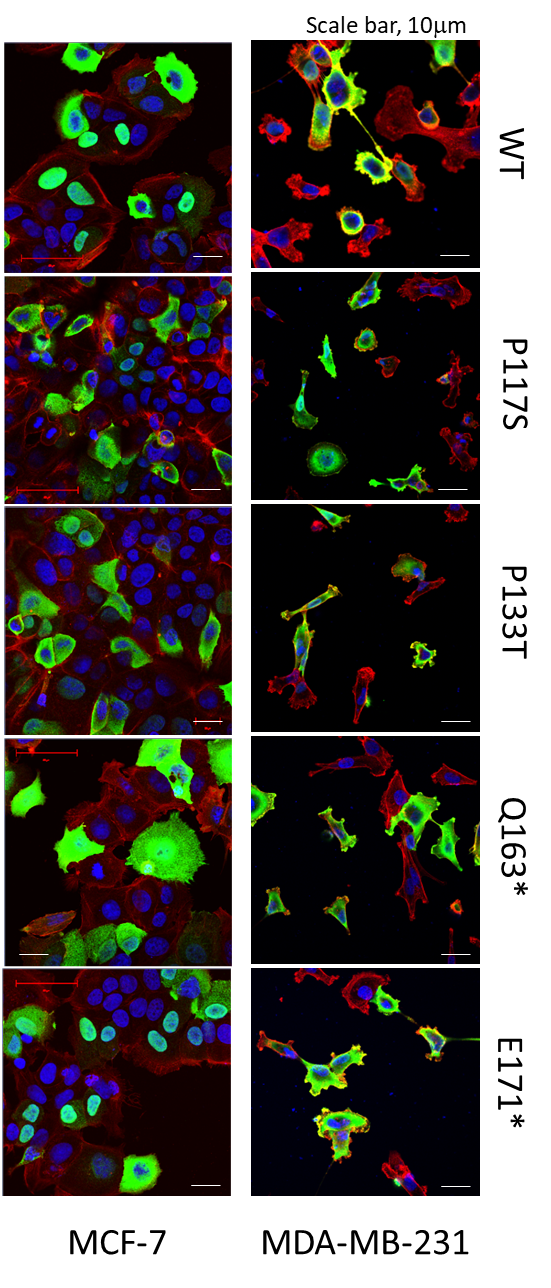


**Figure S1. Immunofluorescence images of cells transfected with p27 WT, missense, and nonsense variants.** MCF-7 and MDA-MB-231 cell lines were grown on coverglasses, and transfected with the indicated plasmids for 24 hours, were processed for immunostaining. Anti-p27 mouse monoclonal/AlexaFluor 488 goat anti-mouse secondary antibodies were used, and DNA was stained by Hoechst. Images were acquired using LSM 700 confocal laser scanning microscope.

**Figure S2**


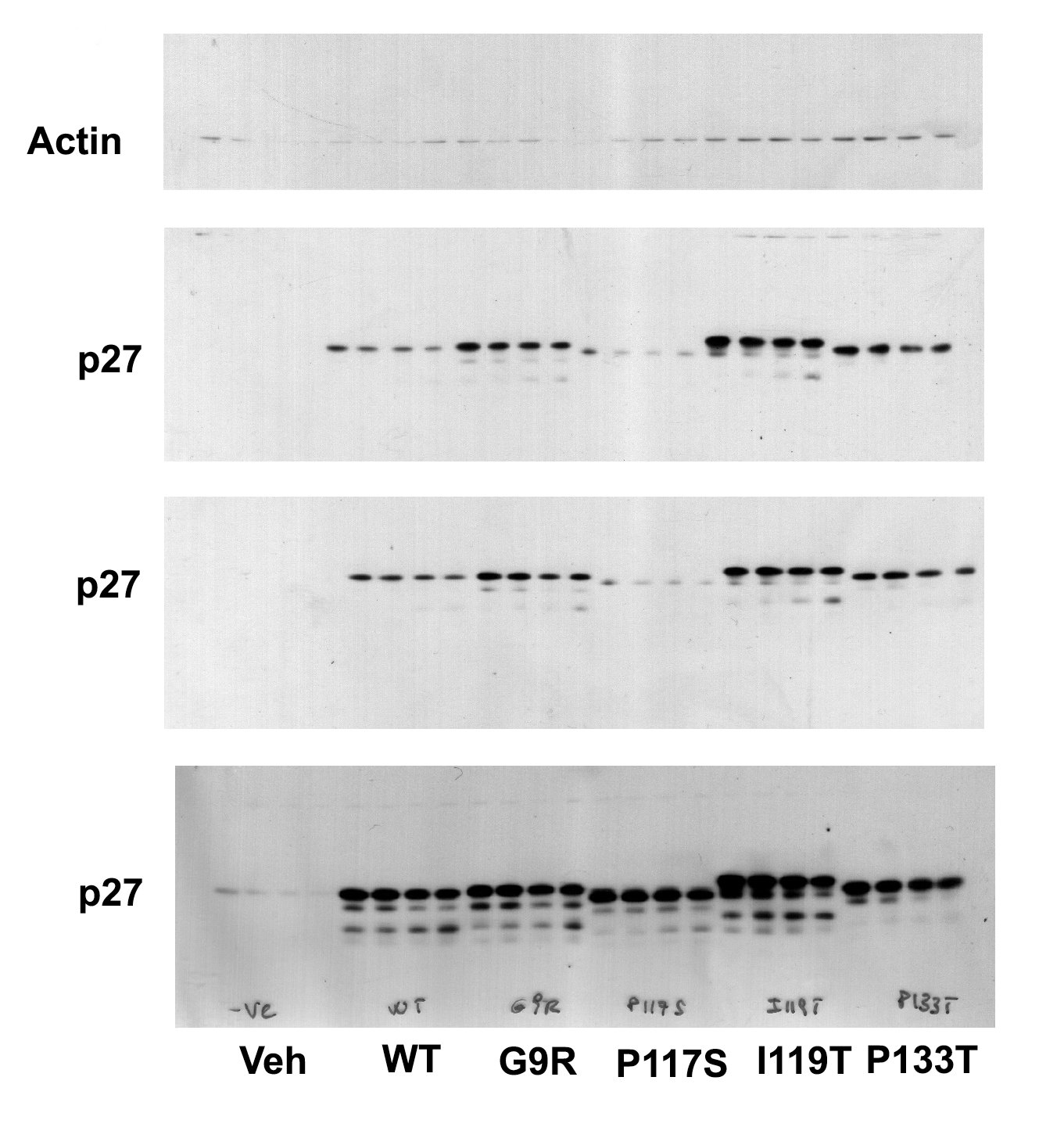


**Figure S2. Original blots for figure 8a (left blots).** The MCF-7 cells were transfected with WT or missense p27 HRVs for 24 h, then they were exposed to 36 μM CHX for 2, 4, and 6 h. At the end of each selected time, cells were collected, total extracts were prepared and analysed through SDS-PAGE/IB with anti-p27 antibody. The Actin was as a loading control. In this experiment, other two missense p27 variants with pathogenic significance were analyzed, i.e. G9R and I119T.

**References**

S1. Bencivenga, D.; Stampone, E.; Aulitto, A.; Tramontano, A.; Barone, C.; Negri, A.; Roberti, D.; Perrotta, S.; Della Ragione, F.; Borriello, A. A cancer-associated CDKN1B mutation induces p27 phosphorylation on a novel residue: a new mechanism for tumor suppressor loss-of-function. *Mol Oncol* **2021**, *15*, 915-941, doi:10.1002/1878-0261.12881.
